# IFN-λ Decreases Murid Herpesvirus-4 Infection of the Olfactory Epithelium but Fails to Prevent Virus Reactivation in the Vaginal Mucosa

**DOI:** 10.3390/v11080757

**Published:** 2019-08-16

**Authors:** Sophie Jacobs, Caroline Zeippen, Fanny Wavreil, Laurent Gillet, Thomas Michiels

**Affiliations:** 1 De Duve Institute, Université catholique de Louvain (UCLouvain), 1200 Brussels, Belgium; 2 Immunology-Vaccinology unit, Department of Infectious and Parasitic Diseases, Faculty of Veterinary Medicine- Fundamental and Applied Research for Animals and Health (FARAH), University of Liège, 4000 Liège, Belgium

**Keywords:** gammaherpesvirus, murid herpesvirus 4, interferon-lambda, type III interferon, respiratory infection, vaginal mucosa, olfactory epithelium

## Abstract

Murid herpesvirus-4 (MuHV-4), a natural gammaherpesvirus of rodents, can infect the mouse through the nasal mucosa, where it targets sustentacular cells and olfactory neurons in the olfactory epithelium before it propagates to myeloid cells and then to B cells in lymphoid tissues. After establishment of latency in B cells, viral reactivation occurs in the genital tract in 80% of female mice, which can lead to spontaneous sexual transmission to co-housed males. Interferon-lambda (IFN-λ) is a key player of the innate immune response at mucosal surfaces and is believed to limit the transmission of numerous viruses by acting on epithelial cells. We used in vivo plasmid-mediated IFN-λ expression to assess whether IFN-λ could prophylactically limit MuHV-4 infection in the olfactory and vaginal mucosae. In vitro, IFN-λ decreased MuHV-4 infection in cells that overexpressed IFN-λ receptor 1 (IFNLR1). In vivo, prophylactic IFN-λ expression decreased infection of the olfactory epithelium but did not prevent virus propagation to downstream organs, such as the spleen where the virus establishes latency. In the olfactory epithelium, sustentacular cells readily responded to IFN-λ. In contrast, olfactory neurons did not respond to IFN-λ, thus, likely allowing viral entry. In the female genital tract, columnar epithelial cells strongly responded to IFN-λ, as did most vaginal epithelial cells, although with some variation from mouse to mouse. IFN-λ expression, however, failed to prevent virus reactivation in the vaginal mucosa. In conclusion, IFN-λ decreased MuHV-4 replication in the upper respiratory epithelium, likely by protecting the sustentacular epithelial cells, but it did not protect olfactory neurons and failed to block virus reactivation in the genital mucosa.

## 1. Introduction

Human gammaherpesviruses (γHVs) are highly seroprevalent and induce an important disease burden, including lymphoid and epithelial cancers. Around 90% of humans worldwide are infected with the Epstein-Barr virus (EBV) [1], and up to 30% with the Kaposi’s sarcoma-associated herpesvirus (KSHV) [2]. Efficient control of those infections would be of public health interest, particularly in regions where co-infection with malaria [3] and human immunodeficiency virus-1 (HIV1) [4] can increase malignancies. Interruption of transmission would be key in the limitation of prevalence. EBV is mainly transmitted via saliva during childhood or adolescence [5]. It is more rarely transmitted via sexual contact [6]. Transmission of KSHV by the saliva is also predominant in populations with a high seroprevalence [7]. In countries where the prevalence is low, increased contact is required for propagation and sexual transmission is more frequent [8,9].

Murid herpesvirus-4 (MuHV-4), formerly also referred to as MHV-68, constitutes a well-described model to study γHV infection and host immune response in the mouse [10]. Like EBV and KSHV, it induces a lytic infection followed by long-term latency in B lymphocytes [11]. After entry in epithelial or neuronal cells, glycoproteins’ conformation changes guide MuHV-4 host colonization from macrophages to dendritic cells, then to B cells in organized lymphoid tissue [12]. Differentiation of submucosal plasma cells allows MuHV-4 reactivation [13] and transfer to overlying epithelial cells, triggering virus re-excretion through saliva and genital fluids [14]. Luciferase imaging of MuHV-4 infection [15] following intranasal (i.n.) inoculation, revealed transient and recurrent genital infection in 80% of female mice from 17 days post-infection (d.p.i.), dependent of the presence of estrogens [16]. Sexual contact with naive males at the time of genital reactivation results in 30% transmission [16]. This model provides a basis to characterize the transmission of γHVs and the therapeutic strategies to limit this transmission.

IFN-λ is induced in response to viral infection and is a key player of the innate immune response at mucosal surfaces (for a recent review, see [17]). It limits the transmission of epitheliotropic viruses by acting on epithelial cells [18,19,20]. Its receptor (IFNLR) is composed of two sub-units: The IFN-λ-specific IFNLR1, and interleukin 10 receptor subunit β (IL10Rβ), which is shared by other IL10-related cytokines [21,22]. Contrary to the type I IFN (IFN-α/β) receptor (IFNAR) which is ubiquitously expressed, IFNLR1 is preferentially expressed by epithelial cells [23]. Some immune cells, such as neutrophils and dendritic cells, have more recently been characterized as IFN-λ responders [24,25,26,27]. Activation of the IFNLR receptor stimulates the Janus kinase - signal transducer and activator of transcription (JAK-STAT) transduction pathway, leading to the phosphorylation of STAT1 and STAT2 that associate with interferon regulatory factor 9 (IRF9) to form a trimeric complex, which triggers the expression of the interferon-stimulated genes (ISGs) [18,28].

Many viruses, however, evolved to evade the IFN response [29]. MuHV-4 has been shown to reduce IFN-α/β induction in infected cells by inhibiting IRF3 via ORF36 [30] and TANK binding kinase 1 via ORF11 [31]. Importantly, it also reduces IFN signaling in a yet-uncharacterized fashion via exonuclease ORF37 [32] and by down-regulating STAT2 via M2 [33]. The virus was also shown to degrade IFNAR via ORF54 [34], while it did not degrade IFNLR1 [35]. The relationship between MuHV-4 and the immune response is complicated by the ability of the virus to spread between different cell types and anatomic sites [14]. The impact of the type I IFN response in the infected host was shown to be cell type-dependent and to determine viral evasion. In the lungs, type I IFNs had a low impact on type I alveolar epithelial cell infection but suppressed lytic macrophage infection, promoting a shift toward latency in B cells [36].

The respiratory route of infection is physiological and well characterized. In the lungs, the virus primarily targets alveolar epithelial cells and alveolar macrophages [37]. In the upper airways, the virus enters the host via olfactory neurons and sustentacular cells before it propagates to the lymphoid organs via myeloid cells [38,39]. In the context of infection by the influenza A virus (IAV), IFN-λ was reported to differentially protect the upper and lower airways. After selective upper airway infections, IFN-λ limited viral shedding from the nasal respiratory epithelium and subsequent spread to the lungs. In the lower airways, IFN-λ alone failed to restrict infection, and a functional type I IFN signaling was required for protection [40].

IFN-α/β therapy against γHVs would offer a narrow window of efficacy, limiting acute infection in myeloid cells [36,39], while IFN-λ could protect epithelial barriers with very few side effects [41,42]. Because transmission of MuHV-4 involves infection of respiratory and/or vaginal epithelial cells, we tested whether IFN-λ could inhibit MuHV-4 transmission. We used in vivo plasmid-mediated IFN-λ expression to assess whether IFN-λ could prophylactically limit MuHV-4 infection in the olfactory and vaginal mucosae.

## 2. Materials and Methods

### 2.1. Animal Experiments

Ethics: Handling of mice and experimental procedures were conducted in accordance with the european community directive 86/609/CEE and the related Belgian law of 6 April 2010. The protocols used in this study were approved by the Committee on the Ethics of Animal Experiments of the University of Liège, under the permit number 1502 of 15 February 2017. BALB/c female mice were housed at the University of Liège, Department of Infectious Diseases, FARAH. B6.A2G-Mx1-IFNAR1^−/−^ female mice were kindly provided by Peter Stäheli (Freiburg University, Freiburg, Germany) [43].

Electroinjection: Mice were electroinjected in the tibialis anterior muscle with the empty vector (pcDNA3) or with IFN-λ2 expression vector (pSJ1) to induce the long-lasting expression of IFN-λ2. General anesthesia was induced with a mix of Medetomidin hydrochlorid 100 mg/mL (Domitor) and Ketamine 5 mg/mL (Anesketin) injected intraperitoneally (i.p.). Before DNA injection, mouse legs were shaved with a depilatory cream. 10 μg of endotoxin free plasmid DNA (pSJ1 or pcDNA3) was injected in 25 μL of phosphate-buffered (PBS) in the tibialis anterior muscle of each hind leg of the mice. Electric pulses (80 V per 4 mm, 8 pulses, 20 ms/pulse, pause: 480 ms) were administered using a Cliniporator system (Cliniporator, IGEA, Carpi, Italy) equipped with 4 mm electrode plates. To ensure electrical contact with the skin, conductive gel was used (Aquasonic 100, ultrasound transmission gel, Parker lab, Fairfield, NJ, USA). After electroinjection, mice were then woken up by an i.p. injection of 250 μL of Atipamezole 500 mg/mL (Antisedan).

Infection and sacrifice: Unless otherwise indicated, 8-week-old BALB/c female mice were infected intranasally with 10^4^ PFU of Luc+ MuHV-4 diluted in 30 μL PBS, under general anesthesia with isoflurane (lower respiratory tract infection), or with 5 × 10^4^ PFU diluted in 5 μL PBS without anesthesia (upper airway infections). They were sacrificed at day 11 or at day 28, and kidneys, spleens and serums were collected. For genital tract collection, the vagina, uterine cervix, and base of the uterine horns were taken jointly.

In vivo luminescence analysis: Mice were injected intraperitoneally (i.p.) with luciferin (60 mg/kg of body weight) and imaged 10 min later with an in vivo imaging system (IVIS) Spectrum instrument (Perkin Elmer, Waltham, MA, USA). For quantitative comparisons, we used Living Image software (Perkin Elmer) to obtain the average radiance (photons per second per square centimeter per steradian) over each region of interest. The background measured in the non-infected right abdominal region was removed from the measurements. The limit of detection of the assay is 10^2^ photons/s/cm^2^/sr.

### 2.2. Viruses

KJ7 and TM967 are Theiler’s murine encephalomyelitis virus (TMEV) (strain DA) derivatives carrying a capsid adapted to infect L929 cells [44]. Codons 5-to-67 of the leader protein coding region were replaced by the open reading frame (ORF) encoding the green fluorescent protein (GFP) (virus KJ7) or the mCherry (virus TM967) fluorescent protein [44]. Those viruses were produced by reverse genetics. The vesicular stomatitis virus (VSV) derivative expressing the GFP was a gift from Martin Schwemmle (University of Freiburg, Freiburg, Germany).

MuHV-4 virus (MHV-68) was derived from a MuHV-4 bacterial artificial chromosome (BAC) [45]. We used a mutant expressing firefly luciferase under the control of an additional M3 (lytic gene) promoter (Luc+ MuHV-4) [15]. The loxP-flanked BAC/enhanced GFP cassette present in the in vitro experiments was removed from the strain used for the in vivo experiments; detailed construction of those variants was previously described [46]. Virus stocks were grown in BHK-21 cells and infected cell supernatants were collected and cleared of cell debris by low-speed centrifugation (1000× *g* for 30 min). Viruses were then concentrated by high-speed centrifugation (58,000× *g* for 90 min). All viruses were titrated by a standard plaque assay on BHK-21 cells.

### 2.3. Cell Culture

The LKR10 cell line (kind gift from Guido Bommer, de Duve Institute, Brussels, Belgium) is derived from lung adenocarcinoma tissues from a K-rasLA1 mouse [47]. BALB/3T3 fibroblasts [48] were kindly provided by Francis Brasseur (Ludwig Institute for cancer research, Brussels, Belgium) and 293T cells [49] by Frédéric Tangy (Pasteur Institute, Paris, France). Those cells, and derivatives, were maintained in Dulbecco modified Eagle medium (DMEM) (Lonza, Vervier, Belgium) containing 4.5 g/L glucose, supplemented with 10% fetal calf serum (FCS) (Sigma-Aldrich, Overijse, Belgium). BHK-21 cells (ATCC) were cultured in Glasgow’s minimum essential medium (GMEM) (Gibco, Thermo Fisher Scientific, Asse, Belgium) supplemented with 10% newborn calf serum and 2.95 g/L tryptose phosphate broth for TMEV and VSV production and for the plaque assay; for MuHV-4 production, it was DMEM (Gibco) supplemented with 2 mM glutamine, and 10% fetal calf serum. All media were supplemented with 50 U/mL penicillin and 50 μg/mL streptomycin (Lonza, Basel, Switherland).

### 2.4. CRISPR Cas 9 Editing

The *Ifnlr1* gene was inactivated in LKR10 cells with the pX461 plasmid (pSpCas9n-2A–GFP) coding for the Cas9 nickase and green fluorescent protein (GFP) [50]. The two single guide RNAs (sgRNAs) were designed using the MIT CRISPR design tool website [51]. Exon 1 of the mouse *Ifnlr1* gene (GenBank: NM_174851) was targeted with two single guides sgRNAs, the combination of which produced no expected off-target cleavage sites during prediction: Annealed oligonucleotides that were cloned at the *Bpi*I sites of pX461 were as follows (guide RNA sequences are underlined): sgRNA1: forward (Fw) 5′-CAC CGA GTA GGG GCG CCC ACC GGT-3′, reverse 5′-AAA CAC CGG TGG GCG CCC CTA CTC-3′; sgRNA2: forward 5′-CAC CGT TCC TGT TGC AGA GCG CCC T-3′, reverse [50] 5′-AAA CAG GGC GCT CTG CAA CAG GAA C-3′. The obtained plasmids targeting *Ifnlr1*, pSJ3, and pSJ4, were co-transfected into LKR10 cells, using TransIT^®^-LT1 transfection reagent (Mirus Bio LLC, Madison, WI, USA), according to the manufacturer’s instructions. After 48 h, GFP-positive cells were sorted by fluorescence activated cell sorting (FACS Aria III, BD biosciences, Franklin Lakes, NJ, USA) and cloned in 96-well plates. Cell clones were screened for loss of type III IFN response with an anti-viral assay. Genome editing of the targeted exon in the clone was further confirmed by sequencing (Genewiz, Essex, United Kingdom).

### 2.5. ISGs and Viral Genome Quantification

ISG expression was measured by RT-qPCR. IFNLR1-KO and WT LKR10 cells were treated with 100 U/mL mouse IFN-αA, 700 pg/mL mouse IFN-λ3, or control supernatant (mock) for 24 h before RNA extraction. Total RNA was extracted from cells, kidney and genital tract using the technique of Chomczynski and Sacchi [52]. Reverse transcription and SybrGreen quantitative PCR (qPCR) for mRNA encoding mouse β-actin, Oasl2 and Usp18 were performed as previously described [53]. Spleen DNA was extracted using Wizard Genomic DNA Purification Kit (Promega, Madison, WI, USA). A Taqman probe was used for MuHV-4 genome quantification (ORF25) by qPCR, as described in [46]. ISG expression and viral genome copy amounts were normalized to those of β-actin, in the cDNA or DNA samples.

### 2.6. Flow Cytometry

For infection analysis, cells were dissociated with trypsin-EDTA and suspended in phosphate-buffered saline (PBS) containing 5% of filtered FCS and 0.5% of paraformaldehyde. Data acquisition was done with an LSR Fortessa flow cytometer (BD biosciences) using FACSDiva software. Data were analyzed using FlowJo 9.6.4. The rate of infection was defined as the percentage of mCherry-or-GFP-positive cells. Viral replication was estimated by the mean of mCherry or GFP fluorescence intensity. Infection efficiency was calculated by combining the rate of infection and viral replication (percentage of mCherry^−/−^ GFP-positive cells multiplied by mean of mCherry/GFP fluorescence of these cells). Fluorescence was measured 8 (VSV) or 24 (TM967, KJ7, MuHV-4) hours post-infection with 0.5 PFU/cell TM967, 0.25 PFU/cell VSV–GFP, 2.5 PFU/cell KJ7 or 1 PFU/cell MuHV-4–GFP.

For cell sorting, transfected (CRISPR-Cas experiments) or transduced (expression experiments) cells were suspended in PBS containing 1% FCS and 1 mM EDTA. GFP-positive cells were cloned at one cell per well in 96 wells plate using the FACS Aria III (BD biosciences, Franklin Lakes, NJ, USA).

### 2.7. Vectors

Plasmids pSJ1 and pSJ7 are pcDNA3 derivatives (Invitrogen, Thermo Fisher Scientific) carrying ORFs of mouse IFN-λ2 and IFN-λ3 [43]. The lentiviral vector pSJ12, used for expression of mouse IFNLR1, was obtained by cloning the Ifnlr1 ORF between the *Bam*HI and *Xba*I sites of pTM945, a lentiviral vector allowing the coexpression of the cloned gene and of mCherry [54]. The *Ifnlr1* ORF sequence was subcloned in this plasmid from pcr2.1-LICR2, kindly provided by Laure Dumoutier (de Duve institute, Brussels, Belgium).

### 2.8. Interferons

Mouse IFN-αA, IFN-λ2 and IFN-λ3 were produced as described previously from 293T cells transfected with pcDNA3-IFN-αA [55], pcDNA3-IFN-λ2 (pSJ1), and pcDNA3-IFN-λ3 (pSJ7). IFNs were diluted in culture medium. Supernatant collected from 293T cells transfected with the empty pcDNA3 vector was used for control treatment of cells and diluted as IFNs. Mouse IFN-λ2 and IFN-λ3 were quantified by ELISA [43]. Mouse IFN-αA anti-viral activity was quantified by a cytopathic effect reduction assay in mouse BALB-3T3 cells, as described previously, using Mengo virus [56].

### 2.9. Immunohistochemistry

Mice were anesthetized before being euthanized for organ harvest and were perfused with PBS-paraformaldehyde (PFA) 1%. Freshly collected heads and genital tracts were fixed in PBS-PFA 4% for 4 h at room temperature. The fixed heads were decalcified in Osteosoft (Merck, Kenilworth, NJ, USA) at room temperature under gentle agitation for 120 h. Organs were then embedded in paraffin. The sections (7 μm) were placed on SuperFrost Plus slides, dried at 37 °C overnight, and processed by standard methods for immunohistochemistry [23]. Sections were blocked with DAKO antibody diluent with background reducing components (S3022, DAKO) for 1 h at room temperature (RT), and then incubated (over-night at 4 °C) with combinations of antibodies to olfactory marker protein (goat PAb; 544-10001 Wako Chemicals, Richmond, VA, USA), anti-cytokeratine-18 (rabbit MAb; NBP2-67468 Novusbio, Centennial, CO, USA), anti-MxA (mouse MAb, #M143 Freiburg university [49]), and E-cadherin (rabbit MAb; #3195 Cell signaling). For immunofluorescent labeling, sections were incubated (for 1 h at RT) with combinations of Alexa 594-conjugated chicken anti-mouse IgG PAb (A-21201, Invitrogen, Carlsbad, CA, USA), Alexa 488-conjugated chicken anti-goat IgG PAb (A-21647, Invitrogen), Alexa 647-conjugated donkey anti-rabbit IgG PAb (A-31573, Invitrogen), Alexa 488-conjugated goat anti-mouse IgG PAb (A-11029, Invitrogen), or Alexa 594-conjugated chicken anti-rabbit IgG PAb (A-21442, Invitrogen), and nuclei were stained with Hoechst 33258 (Sigma Aldrich, Saint Louis, MO, USA). Slides were mounted in Mowiol. Images were captured with a Zeiss LCM510 confocal microscope and analyzed with ImageJ.

### 2.10. Statistics

Statistical analysis was performed using Prism 7 or 8 software (Graph-Pad Software, San Diego, CA, USA). The statistical analysis used and their corresponding *p*-values are indicated in the figure legends.

## 3. Results

### 3.1. IFN-λ Decreases MuHV-4 Infection in Epithelial Cells Overexpressing IFNLR1

**Figure 1:**
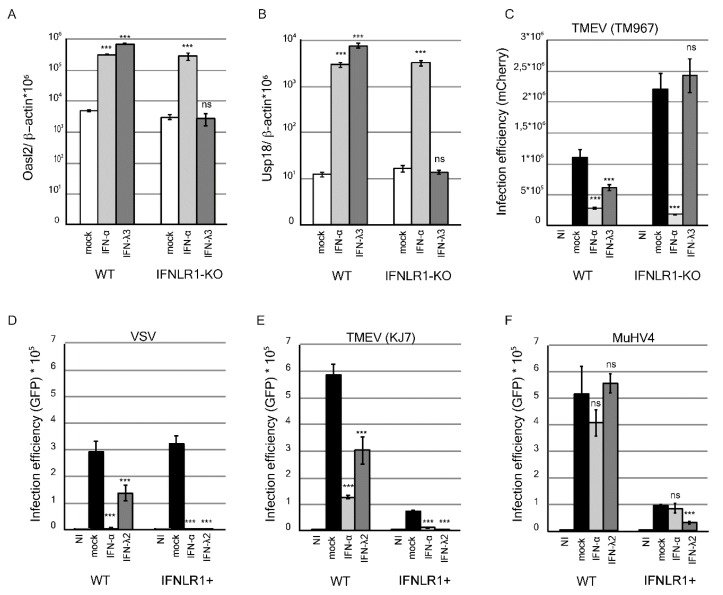
Interferon-stimulated gene (ISG) expression and antiviral activity in response to IFN-λ treatment in WT and IFNLR1-overexpressing LKR10 cells. (**A**,**B**) Amounts of *Oasl2* (**A**) and Usp18 (**B**) per 10^6^ β-actin cDNA copies detected in wild-type (WT) and IFNLR1-KO LKR10 cells 24 h after treatment with mouse IFN-αA, IFN-λ3, or control medium (mock). (**C**) Infection efficiency in WT and IFNLR1-KO LKR10 cells measured by flow cytometry 24 h post-infection with 0.5 PFU/cell of TM967. Cells were pretreated with mouse IFN-αA, IFN-λ3, or mock for 7 h prior to infection. (**D**–**F**) Infection efficiency in WT and IFNLR1-overexpressing (IFNLR1+) LKR10 cells measured by flow cytometry 8 h post-infection with 0.25 PFU/cell vesicular stomatitis virus (VSV)–GFP (**D**); or 24 h post-infection with 2.5 PFU/cell KJ7 (**E**) or 1 PFU/cell murid herpesvirus-4 (MuHV-4)–GFP (**F**). Cells were pretreated with mouse IFN-αA, IFN-λ2 or control medium for 7 h prior to infection. (**A**–**F**) 100 U/mL IFN-αA, 700 pg/mL IFN-λ3, and 1 ng/mL IFN-λ2 were used for treatment. *** *p* < 0.001. ns: non-significant in one-way ANOVA (Dunnett’s test) comparing each group to the mock-treated group.

The inhibitory potential of IFN-λ on MuHV-4 infection was characterized in vitro. In line with their epithelial origin, LKR10 cells were shown to express both type I and type III IFN receptors [43]. In these cells, mouse IFN-αA and IFN-λ3 treatments significantly up-regulated the expression of the IFN-stimulated genes (ISGs) *Oasl2* (Figure 1A) and *Usp18* (Figure 1B), and significantly decreased infection by TM967, a TMEV derivative expressing mCherry (Figure 1C). As expected, IFN-λ-mediated ISG induction and anti-viral activity were abolished in an LKR10 cell clone in which the IFNLR1 receptor subunit had been knocked-out (Figure 1A–C).

Next, we assessed the antiviral activity of IFN-λ against MuHV-4. After treatment with 1 ng/mL IFN-λ2, replication of vesicular stomatitis virus (VSV)–GFP and TMEV–GFP (KJ7) was significantly reduced but not that of MuHV-4–GFP (Figure 1D–F, graphs on the left). Antiviral activity of IFN-λ against VSV and TMEV was, however, moderate as compared to that of IFN-αA (Figure 1D,E), suggesting that LKR10 cells expressed only moderate amounts of the receptor. We thus tested the antiviral activity of IFN-λ in LKR10 cells that overexpress IFNLR1, in which the response to IFN-λ was shown to be enhanced [43]. In such LKR10-IFNLR1+ cells, IFN-λ2 treatment inhibited VSV and KJ7 (Figure 1D,E, right graphs) infection by more than 95%, while MuHV-4 infection was decreased by 65% (Figure 1F, right). Of note, cells overexpressing IFNLR1 likely exhibit increased basal STAT1 expression levels and behave as “IFN-primed” cells, as was observed previously in Fawa-λ-luc cells [43]. This may explain the lower infection efficiency observed in these cells with TMEV and MuHV-4. Why VSV infection efficiency was not affected is however unclear.

Taken together, these data show that MuHV-4 exhibits some resistance to IFN-λ, as previously shown for IFN-α in vivo [36]. MuHV-4 infection can, however, be reduced in cells that express high levels of the receptor.

### 3.2. IFN-λ Decreases Nasal but not Lung MuHV-4 Infection

We hypothesized that, in vivo, IFN-λ might protect some epithelial cells from MuHV-4 infection because these cells respond readily to type III IFNs. Using the Luc+ MuHV-4 strain that expresses the firefly luciferase gene, we analyzed the impact of IFN-λ on the primary infection of the respiratory tract using luminescence with an in vivo imaging system (IVIS).

**Figure 2:**
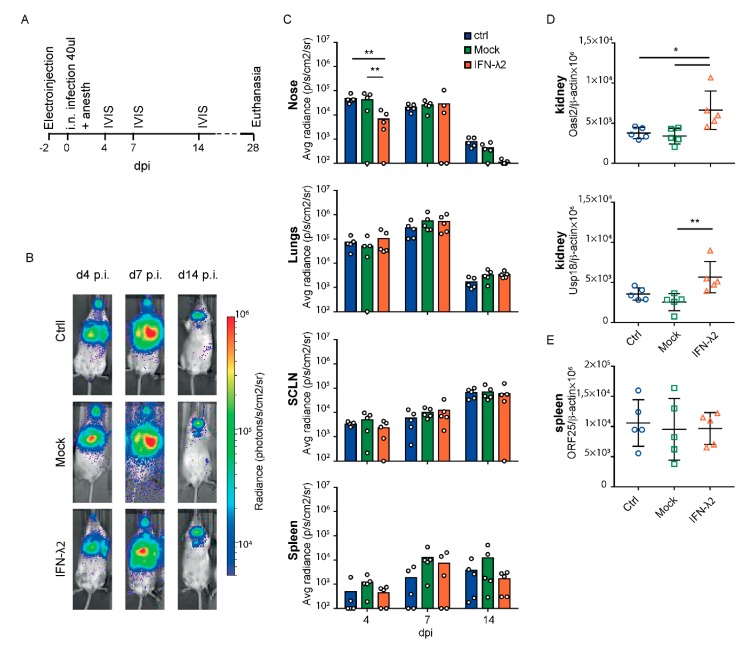
Bioluminescence analysis of MuHV-4 after intranasal inoculation under general anesthesia in mock or IFN-λ-treated mice. (**A**) Eight week-old female BALB/c mice were electroinjected with an empty plasmid (mock, *n* = 5) or a plasmid expressing IFN-λ2 (IFN-λ2, *n* = 5). A control group (ctrl, *n* = 5) was left untreated. Two days later, mice were infected intranasally with 10^4^ PFU Luc+ MuHV-4 in 40 μL under general anesthesia and imaged at the times indicated. (**B**) Representative images are shown for ctrl, mock or IFN-λ2 electroinjected groups at days four, seven, and 14 post infection (p.i.) (**C**) Signal intensities were compared for the nose, lungs, superficial cervical lymph nodes (SCLNs) and spleen between groups. The signal was determined by measuring equivalent regions of interest and subtracting the right abdominal signal as a negative background. The graphs show mean and individual values. (**D**) Amounts of *Oasl2* and *Usp18* per 10^6^ β-actin cDNA copies detected in the left kidney of mice at the time of sacrifice. (**E**) Viral genome copy numbers (ORF25) per 10^6^ β-actin copies in spleen at the time of sacrifice showed no significant difference between groups. (**C**–**E**) Two-way (**C**) and one-way (**D**,**E**) ANOVA: * *p* < 0.05 and ** *p* < 0.01; no mark—no significant difference.

Intranasal inoculation of mice under general anesthesia induces concomitant upper and lower airway infections. Two days before Luc+ MuHV-4 infection, mice were electroinjected with either an empty plasmid or a plasmid (pSJ1) allowing the long-lasting production of circulating mouse IFN-λ2. This technique of plasmid electroinjection has the advantage of leading to long-lasting circulating IFN production without the need for repeated injections, which might influence the inflammatory response. A control group was left untreated (Figure 2A). Four d.p.i., a significant decrease of signal was observed in the snout of IFN-λ2-treated mice as compared to untreated or empty vector-administered mice. In contrast, the signal was comparable in the lungs of all three groups, as in other infected organs up to 14 d.p.i (Figure 2B,C). Virus replication (i.e., luciferase signal) in the nose and the lungs of all mice then dropped between 7 and 14 d.p.i. while it increased in lymph nodes, in agreement with previous reports showing that type I IFN gradually suppressed alveolar macrophage infection but failed to prevent B cell infection where MuHV4 would evade the IFN response [36,37].

Long term systemic IFN-λ production from the electroinjected plasmid was confirmed by a significant upregulation of the ISGs *Oasl2* and *Usp18* in the kidneys of the IFN-λ2-treated mice, 28 days post-treatment (Figure 2D). Quantification of the viral genome by qPCR confirmed that the viral load was not modified in the spleen by the IFN-λ treatment (Figure 2E). As the mock-treated group did not significantly differ from the controls, we concluded that the electroinjection process itself did not influence the infection profile.

In conclusion, IFN-λ expression decreased infection of the snout early after intranasal infection but did not prevent progression of the virus to the lungs, the lymph nodes, and the spleen.

**Figure 3:**
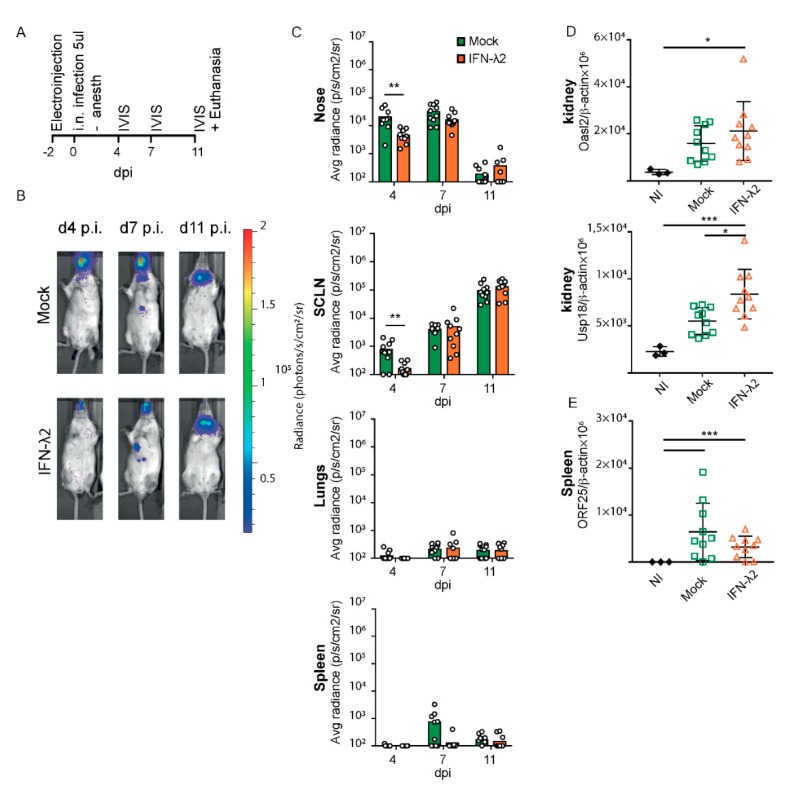
Bioluminescence analysis of MuHV-4 after intranasal inoculation without anesthesia in mock or IFN-λ-treated mice. (**A**) Eight week-old female BALB/c mice were electroinjected with an empty plasmid (mock, *n* = 10) or a plasmid expressing IFN-λ2 (IFN-λ2, *n* = 10). Two days later, alert mice were infected by intranasal (i.n.) injection with 5 × 10^4^ PFU Luc+ MuHV-4 in 5 μL and imaged at the times indicated. (**B**) Representative images are shown for mock or IFN-λ2 electroinjected groups at days four, seven, and 11 p.i. (**C**) Signal intensities were compared for the nose, superficial cervical lymph nodes (SCLNs), lungs, and spleen between groups. (**D**) Amounts of *Oasl2* and *Usp18* transcripts per 10^6^ β-actin copies detected in the left kidney of non-infected (NI, *n* = 3), mock, or IFN-λ2-treated mice at the time of sacrifice. (**E**) Viral genome copy numbers (ORF25) per 10^6^ β-actin copies in spleen at the time of sacrifice showed no significant difference between groups. (**C**) Student’s t-test was used to compare mock and IFN-λ-treated mice. In this analysis, one outlier defined by a Grubs’ test (*p* < 0.01) was ignored, which strengthened the significance from * to **. (**D,E**) One-way ANOVA: * *p* < 0.05, ** *p* < 0.01 and *** *p* < 0.001; no mark—no significant difference.

We then analyzed the effect of IFN-λ expression on the upper airway infections, by infecting alert mice with a reduced inoculum volume. This virus inoculation method was reported to favor upper airway infections. As above, mice were electroinjected two days prior to infection, with the empty or the IFN-λ2-expressing plasmid (Figure 3A). Luciferase activity was followed at 4, 7 and 11 d.p.i., the latter time point allowing clear detection of the signal in SCLNs after infection of the upper airways [15].

A small but significant decrease of the luciferase signal was observed in the snout and superficial cervical lymph nodes of the IFN-λ plasmid-treated mice 4 d.p.i. (Figure 3B,C). As expected after infection of alert mice, no signal was observed in the lungs and the virus propagated directly from the nose to the lymphoid organs. Thirteen days post-treatment, ISGs were significantly more induced in mice that received the IFN-λ plasmid than in mice that received the empty vector (Figure 3D). Given the lytic infection present 11 d.p.i. that induces IFN production, some extent of ISG induction was observed in the mock-treated group that had been infected but ISG induction was not as strong as in IFN-λ plasmid-treated mice. The viral load in the spleen 11 d.p.i did not significantly differ between groups (Figure 3E).

Thus, after infection of both anesthetized and alert mice, prophylactic IFN-λ expression reduced early MuHV-4 infection in the upper respiratory tract but not in the lungs or spleen.

### 3.3. Sustentacular Cells of the Olfactory Epithelium Respond to IFN-λ Whereas Olfactory Neurons Do Not

**Figure 4:**
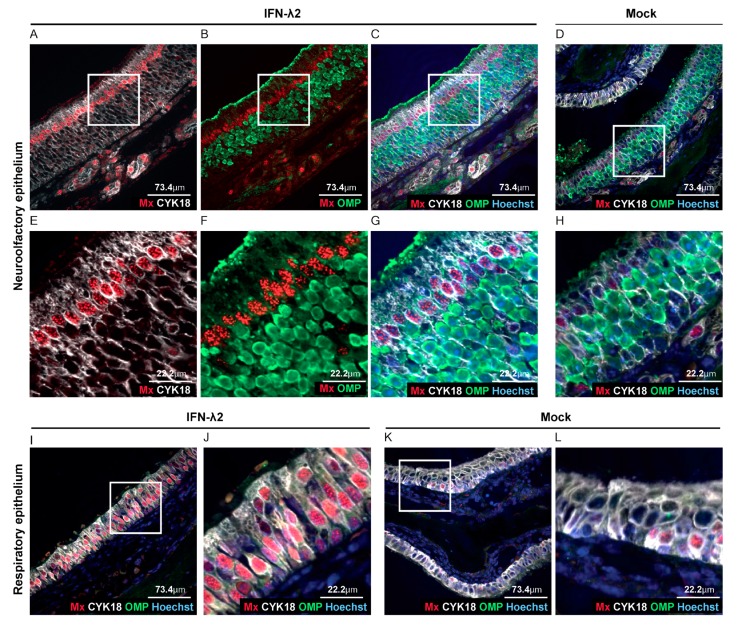
IFN-λ responsive cells in the olfactory epithelium. (**A**–**L**) Representative sections of the olfactory and respiratory nasal epitheliums labeled for Mx1 (red), olfactory marker protein (OMP) (neurons, green) and CYK18 (sustentacular cells, white). Where indicated, nuclei were stained with Hoechst dye (blue). Sections were from B6.A2G-Mx1-IFNAR1^−/−^ female mice, seven days after electroinjection of an empty plasmid (*n* = 4) or a plasmid expressing IFN-λ2 (*n* = 4). (**A**–**C**,**E**–**G**) Representative sections of the olfactory epithelium of IFN-λ plasmid-treated mice revealing extensive Mx1 staining in sustentacular cells but not in neurons. (**D**,**H**) Little Mx1 expression in the olfactory epithelium of mock-treated mice. (**E**–**H**) Images zoomed-in from the upper panel (white square). (**I**–**L**) Extensive Mx1 expression in the respiratory epithelium of IFN-λ-treated mice (**I**–**J**) compared to mock-treated mice (**K**,**L**). (**J**,**L**) Images zoomed-in from the corresponding left panels (white square).

After i.n. inoculation of alert mice, Luc+ MuHV-4 was shown to infect olfactory neurons and sustentacular cells of the olfactory epithelium, but not respiratory epithelial cells [38,39]. While the respiratory epithelium had been shown to respond to IFN-λ [40], response to IFN-λ in the olfactory epithelium had not been characterized. We thus analyzed Mx1 expression as a marker of IFN response, in head sections of B6.A2G-Mx1-IFNAR1^−/−^ mice that were electroinjected with an empty vector or an IFN-λ2-expressing plasmid. Co-staining of neurons (olfactory marker protein—OMP), sustentacular cells (cytokeratine 18—CYK18) and Mx1 protein revealed that all luminal sustentacular cells (CYK18+/OMP−) were responsive to IFN-λ, while the underlying OMP+ neurons were not (Figure 4A–H). The respiratory epithelium was strongly Mx1-positive in IFN-λ-expressing mice (Figure 4A–C,E–G,I–J) whereas, as expected, only few sustentacular and respiratory epithelial cells were Mx1-positive in mock-treated mice (Figure 4D,K–L).

Taken together, our results suggest that the decreased infection of the upper airways observed at day four post infection (Figure 2B,C and Figure 3B,C) correlates with the protection of sustentacular cells of the epithelium by IFN-λ. The absence of the protection of downstream virus target organs, such as the spleen, thus likely stems from the direct infection of olfactory neurons that exhibited no responsiveness to IFN-λ.

### 3.4. IFN-λ Expresion Fails to Prevent Genital Reactivation from Latency in Female Mice

Local IFN-λ treatment has been shown to protect the mouse vaginal epithelium from herpes simplex virus (HSV)-2 infection [18]. We hypothesized that prophylactic vaginal epithelium exposure to IFN-λ might prevent genital reactivation of MuHV-4 and subsequent transmission to partners.

**Figure 5:**
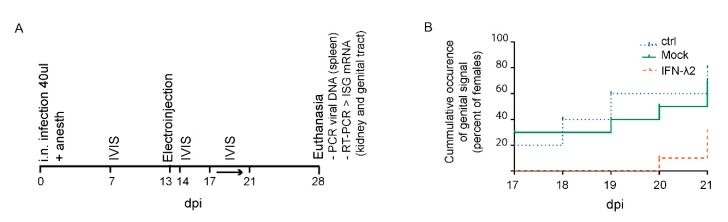
Luciferase signal occurrence in the genital tract of IFN-λ expressing mice. (**A**) Outline of the experiment: Eight week-old BALB/c female mice were infected by i.n. injection with 10^4^ PFU Luc+ MuHV-4 under general anesthesia. Thirteen d.p.i., mice were electroinjected with an empty plasmid (mock, *n* = 10) or a plasmid expressing IFN-λ2 (IFN-λ2, *n* = 10). A control group (ctrl, *n* = 10) was left untreated. To assay the influence of IFN-λ on genital tract colonization, mice were imaged every day from day 17 to 21 p.i. After sacrifice, upregulation of IFN-stimulated gene (ISG) induction was followed by RT-PCR and spleen infection was detected by PCR. (**B**) Non-significant difference observed between groups in the occurrence of a genital signal from day 17 to 21 p.i. non-significant difference by Log Rank test (Mantel–Cox): *p* > 0.05.

In a preliminary experiment, three groups of BALB/c female mice (*n* = 10) were infected intranasally with Luc+ MuHV-4 under general anesthesia. After establishment of latency, a control group was left untreated while the two others were electroinjected with either an empty or an IFN-λ2-expressing plasmid (Figure 5A). IVIS measurements at 7 and 14 d.p.i. confirmed the expected propagation of the virus from the lungs to the lymphoid organs in all mice. IFN-λ expression in the group that received the IFN-λ2 plasmid was confirmed by a significant ISG upregulation in the kidney and in the genital tract at 28 d.p.i.. Infection of mice was confirmed by viral genomic DNA detection in the spleen at the time of sacrifice.

Genital signal occurrence (i.e., virus reactivation) and intensity was then recorded from day 17 to day 21 p.i. This five day follow-up coincides with the estrous cycle of the mouse and should include an estrogen peak accompanied by virus reactivation in the genital tract of most of the mice [16]. The mock-treated group (7/10 reactivations) did not significantly differ from the untreated controls (8/10 reactivations) (Figure 5B). In the IFN-λ2 plasmid-treated group, a non-significant reduction of signal occurrence was observed (3/10 reactivations). Moreover, the genital signal in the IFN-λ2 expressing mice was only observed in the last days of follow-up, while in the other groups genital signal appeared randomly along the week (Figure 5B). These data suggested that IFN-λ2 may reduce or delay virus reactivation in the genital mucosa, though non-significantly.

**Figure 6:**
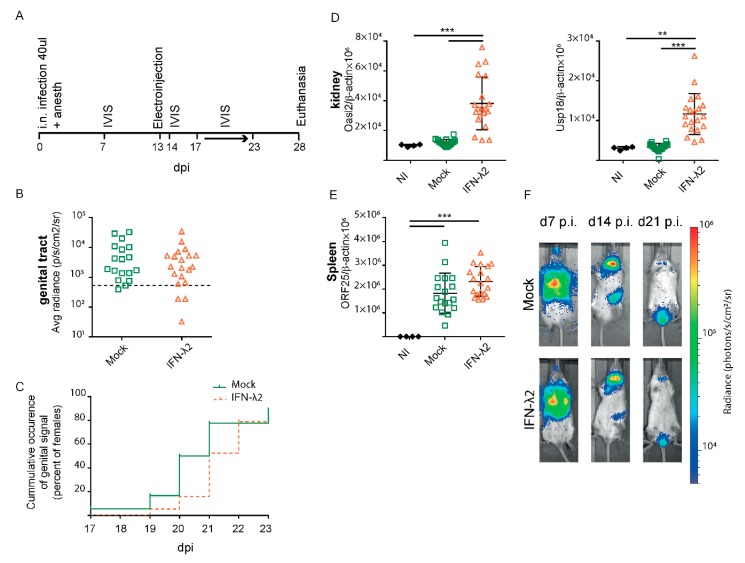
MuHV-4 reactivation in the genital tract despite IFN-λ treatment. (**A**) Same experiment as in Figure 5 with mock (*n* = 20) and IFN-λ2-treated (*n* = 20) mice and an extended luciferase imaging time to assess a potential delay in the genital reactivation. (**B**,**C**) No significant difference was observed between groups in the maximal genital signal (**B**) and in the time of occurrence of a genital signal (**C**) from day 17 to 23 p.i. Positive signals were taken as those that were >2 standard deviations above the mean for 10 uninfected mice (threshold represented by the dashed line). (**D**) Amounts of *Oasl2* (left) and *Usp18* (right) transcripts per 10^6^ β-actin copies detected in the left kidney of non-infected (NI, *n* = 4), mock, or IFN-λ2-treated mice at the time of sacrifice. (**E**) Viral genome copy numbers (ORF25) per 10^6^ β-actin copies in spleen at the time of sacrifice showed a significant difference between groups. (**F**) Representative images are shown for mock or IFN-λ2 electroinjected groups at days seven, 14, and 21 p.i. (**B**,**D**,**E**) Student’s *t*-test (**B**) and one-way ANOVA (D–E): *p* < 0.01 and *** *p* < 0,001; no mark—no significant difference. (**C**) Log Rank test (Mantel–Cox): *p* > 0.05.

To confirm whether IFN-λ expression was able to decrease or to delay the genital infection, the experiment was reproduced with groups of 20 mock- and IFN-λ2-treated mice. IVIS measures were performed at 7 and 14 d.p.i. to confirm the usual evolution of infection. In this experiment, the reactivation follow-up period was extended up to 23 d.p.i. to detect a potentially delayed genital reactivation (Figure 6A). No reduction in signal frequency and intensity was observed, however, after IFN-λ2 plasmid-treatment (Figure 6B). Again, a non-significant delay of one day was observed in the appearance of the signal (Figure 6C). Upregulated ISGs expression in the kidneys confirmed IFN-λ expression from the administered plasmid (Figure 6D). Viral genome loads were detected by PCR in the spleen of all mice (Figure 6E), and were surprisingly slightly higher in the spleen of the IFN-λ expressing mice.

**Figure 7:**
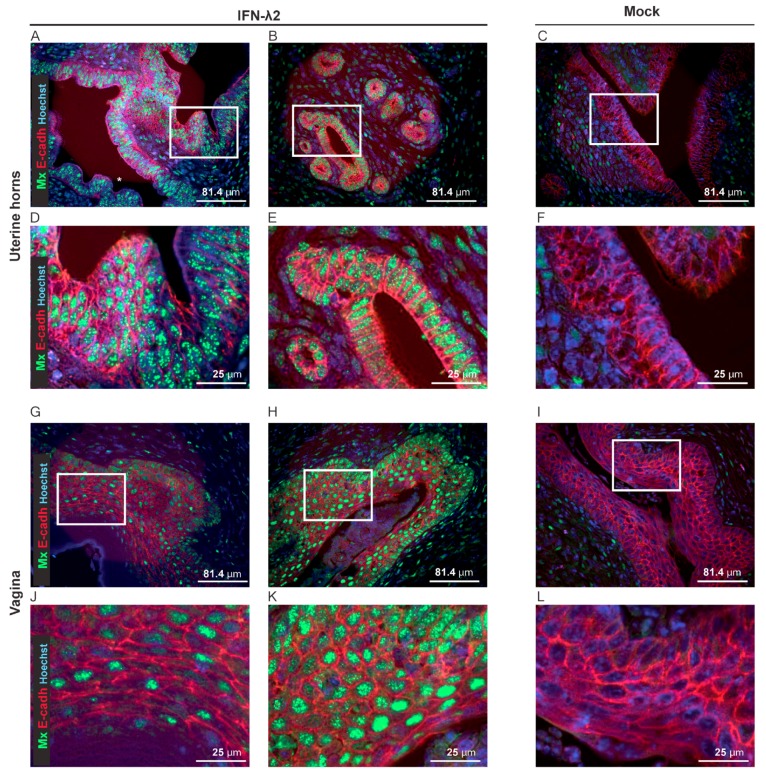
IFN-λ-responsive cells in the genital tract. (**A**–**L**) Representative sections of the uterine horns and vaginas showing Mx1 expression (green) and E-cadherin+ epithelial cells (E-cadh, red) seven days after electroinjection of B6.A2G-Mx1-IFNAR1^−/−^ female mice with an empty plasmid (Mock, *n* = 4) or a plasmid expressing IFN-λ2 (*n* = 4). (**A**–**F**) Representative sections of the uterine horns revealing extensive Mx1 expression in the E-cadherin positive cells of two IFN-λ-treated (**A**,**B**) compared to the mock-treated (**C**) mice. (**D**–**F**) Images zoomed-in from the upper panel (white rectangle). (**G**–**L**) Representative sections of the vaginal epithelium revealing partial (**G**–**J**) versus extensive (**H**–**K**) Mx1 expression in the E-cadherin positive cells of two IFN-λ-treated mice and little Mx1 expression in the E-cadherin positive cells of mock-treated mice (**I**–**L**). (**J**–**L**) Images zoomed-in from the upper panel (white rectangle).

To characterize the IFN-λ response of the vaginal epithelial cells, Mx1 immunostaining was performed on genital tract sections of B6.A2G-Mx1-IFNAR1^−/−^ mice, seven days after electroinjection of empty or IFN-λ2-expressing plasmids. Columnar epithelial cells of the uterine horns strongly responded to IFN-λ (Figure 7A–F). In the vaginal epithelium, the majority of the e-cadherin+ epithelial cells were Mx1-positive after IFN-λ treatment, but variation occurred between mice (Figure 7G–L).

In conclusion, IFN-λ non-significantly delayed genital reactivation of MuHV-4 infection despite extensive IFN-λ response in the epithelium of the uterine horns and a more variable response in the vaginal epithelium.

## 4. Discussion

Viral transmission is the condition for virus evolution and maintenance in the human population. Herpesviruses have a high prevalence that could be decreased by targeting their transmission. To infect new hosts, viruses need to disrupt or cross the epithelial barriers. IFN-λ is a key actor of the innate immune response that limits infection of viruses targeting epithelial cells. In the mouse digestive tract, IFN-λ limits norovirus shedding [19] and restricts host-to-host transmission [20] by acting on epithelial cells. Influenza transmission to naive contact was also restricted by IFN-λ, which protects the upper airways [40]. In the genital tract, prophylactic intra-vaginal IFN-λ treatment prevented HSV-2 infection in the vaginal epithelium of mice [18]. Although herpesviruses developed many strategies to escape the IFN response and persist, increasing the antiviral defense via IFN-λ treatment might tip the scale in favor of the host. γHVs escape the innate immune response and establish latency, mainly in B lymphocytes, a conserved pattern among the species [14]. Transmission between hosts occurs via re-excretion of the virus through epithelial cells. IFN-λ administration might thus prevent spreading of the virus by protecting the epithelial barrier, with limited side effects [41,42]. Here we analyzed the effect of IFN-λ on infection by MuHV-4, the mouse model for γHVs infection.

In vitro, IFN-λ treatment decreased MuHV-4 infection only when cells overexpressed the IFN-λ receptor. Antagonism of MuHV-4 infection was, however, less effective than that of VSV or TMEV. As was shown for type I IFNs [36], MuHV-4 partly resists IFN-λ signaling, but infection can be dampened in cells that express high levels of the receptor. As IFNLR1 is readily expressed by epithelial cells in vivo [23], we hypothesized that prophylactic IFN-λ treatment of mice might protect the respiratory and genital epitheliums from MuHV-4 infection and limit its transmission.

We first tested the antiviral effect of IFN-λ treatment on the primary infection of the respiratory tract and tracked the infection by bioluminescent analysis. Prophylactic IFN-λ treatment partially reduced the nasal luminescent signal, but no difference was seen in the lungs. The absence of lung protection in our experiments is in agreement with previous studies stating that alveolar macrophages, which are not expected to mount a strong IFN-λ response are the first cells infected in the lung [37].

After selective upper airway infections, the nasal and superficial lymph nodes’ signal was reduced at 4 d.p.i. in IFN-λ-treated mice, but not at later time points. In the nasal cavity, infection of the olfactory epithelium by MuHV-4 has been well documented [38,39], and involvement of the respiratory epithelium was observed in IFNAR^−/−^ mice [39]. Type I IFN has been shown to be instrumental in the protection of the olfactory bulb after VSV infection. Type I IFN produced by neuroectodermal cells and primarily by astrocytes activated microglial cells [57] and protected distal parts of the brain [58]. Regarding the type III IFN response, the influence of IFN-λ on respiratory epithelial cells to IFN-λ is well documented [40] but little is known about the response of olfactory neurons and sustentacular cells. The nasal luciferase signal (i.e., MuHV-4 replication) reduction observed at early time points after IFN-λ treatment indicates that some of the MuHV-4 target cells are responsive to type III IFNs. Mx1 immunostaining of B6.A2G-Mx1-IFNAR1^−/−^ IFN-λ-treated mice revealed that luminal sustentacular cells are strongly responsive to IFN-λ, while underlying olfactory neurons are not. It is thus likely that in IFN-λ-treated mice, the observed MuHV-4 infection delay reflected the resistance of sustentacular cells and that infection occurred through the unprotected olfactory neurons.

Sexual transmission of MuHV-4 was documented and follows transient virus reactivation in the vaginal mucosa of infected female mice [16]. Preventing viral shedding from the infected host would constitute an interesting therapeutic option to limit sexual transmission. Bioluminescent analysis of female mice treated with IFN-λ after the establishment of latency, revealed a non-significant delay in luciferase signal appearance, and no decrease in the intensity of the genital signal. In genital tract sections of IFN-λ-treated B6.A2G-Mx1-IFNAR1^−/−^, columnar epithelial cells of the uterine horns strongly and consistently expressed Mx1. Vaginal epithelial cells were also responsive to IFN-λ but the response was less consistent between mice. This variability in the response of the vaginal epithelium was not due to ineffective IFN-λ expression given the strong Mx1 induction observed in other epithelial tissues, such as the uterine horns and olfactory epithelium of the same mice. Variability in Mx1 expression in the vaginal epithelium likely correlated with variation in the estrus cycle, as suggested by epithelial thickness differences. Vaginal epithelium differentiation and proliferation are known to be under hormonal control and to vary with the estrus cycle [59,60]. Some studies suggest that estrogens dampen the innate immune response in the female genital tract [58,61]. On the other hand, Caine et al. recently showed that in ovariectomized mice, IFN-λ administration was antiviral against Zika virus when mice were treated with estradiol and progesterone (pro-estrus stage) but not with progesterone only (diestrus) [62]. The hormonal status of the mouse thus influences the response to IFN-λ. Genital reactivation of MuHV-4 infection was reported to occur during the estrus phase of the cycle [16]. A decrease in IFNLR1 expression or signaling, at the time where the epithelial cells are permissive to MuHV-4 infection, might contribute to the viral escape to IFN-λ treatment.

In conclusion, MuHV-4 can escape to some extent, the control by IFN-λ. In vitro, MuHV-4 was less affected by IFN-λ treatment than VSV or TMEV. In vivo, MuHV-4 likely bypasses the IFN-λ response by infecting unresponsive cell types, such as neurons in the olfactory epithelium. In vaginal epithelial cells, MuHV-4 may take advantage of a transient decrease in the IFN-λ response during the estrus cycle.
